# Grit, Self-Efficacy, Motivation and the Readiness to Change Index Toward Exercise in the Adult Population

**DOI:** 10.3389/fpsyg.2021.732325

**Published:** 2021-08-12

**Authors:** Manuel De La Cruz, Alex Zarate, Jorge Zamarripa, Isabel Castillo, Angelica Borbon, Hector Duarte, Kathryn Valenzuela

**Affiliations:** ^1^Programa Educativo Licenciado en Entrenamiento Deportivo, Universidad Estatal de Sonora, Hermosillo, Mexico; ^2^Facultad de Organización Deportiva, Universidad Autónoma de Nuevo León, San Nicolás de los Garza, Mexico; ^3^Department of Social Psychology, University of Valencia, Valencia, Spain

**Keywords:** grit, self-efficacy, motivation, stages of change, exercise

## Abstract

This study examined the relationships between grit personality, self-efficacy, motivation (autonomous, controlled, and amotivation), and the readiness to change index toward exercise. Participants were 391 adults aged between 18 and 64 years old (*M* = 31.16; *SD* = 12.45) from Hermosillo, Sonora (Mexico) who completed questionnaires (i.e., the Grit Personality Scale, the Exercise Self-Efficacy Questionnaire, the Treatment Self-Regulation Questionnaire and the Stages of Change Questionnaire for Physical Activity) measuring the variables of interest. The reliability of the instruments was tested using Cronbach's alpha, whereas confirmatory factor analyses were performed for each instrument separately. A measurement model and a structural equation model were assessed as well. The results of the structural equations model showed that grit personality was positively associated with self-efficacy, and in turn, with autonomous motivation and with the readiness to change index. On the other hand, self-efficacy was negatively correlated with controlled motivation, and positively correlated with the readiness to change index. Finally, self-efficacy also showed a negative correlation with amotivation, which, in turn, was negatively correlated with the readiness to change index. These results provide information to develop psychological intervention programs based on grit personality and motivation, with the aim of increasing the number of participants who engage in exercise.

## Introduction

Most people acknowledge that maintaining a sedentary lifestyle may lead to negative consequences on health and life quality in the long term, and that regularly engaging in physical activity provides health benefits and prevents medical complications. Nevertheless, the World Health Organization (WHO) has underscored that only few people are successful in overcoming sedentary behaviors, resulting in the high levels of sedentary lifestyles seen around the world (World Health Organization, 2018). In some cases, physical inactivity (i.e., insufficient physical activity level to meet present physical activity recommendations; e.g., adults ≥18 years, not achieving 150 min of moderate-to-vigorous-intensity physical activity per week, World Health Organization, 2018), and low activity adherence stem from psychological factors (Andia et al., 2014; Fernández-Ozcorta et al., 2016). Grit personality is a construct that has gained relevance in the study of maintaining healthy behaviors (Duckworth et al., 2007; Reed et al., 2013). This construct refers to the individual's continuous effort and interest to achieve a long-term goal in life. Grit personality is defined as the perseverance and passion to reach goals attainable after years of work. This leads to being able to work determinedly toward challenges, maintaining the same effort and interest throughout the years despite the setbacks along the way (Duckworth et al., 2007).

The grit personality has been associated with self-efficacy. Self-efficacy is defined as the belief that people have about their own ability to organize and execute the necessary courses of action to attain specific achievements (Bandura, 1977), for example, modifying a behavior or conduct with regard to a particular habit. Self-efficacy theory suggests that control and personal action are two crucial factors for the individual to develop self-efficacy (Bandura, 1977). In other words, an individual seeking to work on self-efficacy must first convince themselves that they are in control of the situation and that their actions uphold an intention and a motivation. In a recent study, Ciaccio (2019) explored the relationships between grit and its dimensions (perseverance of effort and consistency of interest), and self-efficacy toward exercise in 366 university students. Results revealed that grit and perseverance of effort were positively correlated with self-efficacy toward exercise, whereas consistency of interest was not correlated. This suggests that both constructs can work together for the adoption and maintenance of healthy behaviors in people.

Motivation, on the other hand, is another variable that has been widely employed to explain behaviors toward exercise. According to Deci and Ryan (1985), motivation conveys the energy (intensity) and direction (giving meaning to internal and external stimuli) of behavior. In Self-Determination Theory (SDT; Deci and Ryan, 1985; Ryan and Deci, 2017), motivation is seen as a gradient that varies according to the level of volition or self-determination of behaviors, where the most and the least self-determined forms are intrinsic motivation and amotivation, respectively. Between these two forms of motivation is extrinsic motivation, which has different types of regulations depending on their level of self-determination: integrated regulation, identified regulation, introjected regulation and external regulation. The most self-determined motivation is intrinsic motivation, characterized by behaviors exercised for the sole enjoyment or pleasure inherent to the activity. Integrated regulation is characterized because the behavior is carried out freely; the individual evaluates the behavior and acts in accordance with their principles and needs. Identified regulation refers to a conscious assessment of an objective or behavior; this is related to aspects that are relevant to the individual: “I must exercise because it is important to me.” Introjected regulation is related to expectations of self-approval, ego enhancement and pride. External regulation refers to an external demand, expecting some reward or avoiding some punishment; this is the lowest self-determination level for extrinsic motivation. Lastly, amotivation is the mere absence of all the aforementioned (Deci and Ryan, 2000).

SDT indicates that these regulations can be grouped in a broader sense to form autonomous motivation vs. controlled motivation. Autonomous motivation stems from an internal feeling where the person shows the need to explore the environment, and the curiosity and the pleasure experienced from carrying out an activity for the mere sake of doing it. On the other hand, controlled motivation is conditioned by an external factor or feeling (Deci and Ryan, 2000). Studies have examined the relationship between self-efficacy and the different types of motivation toward physical activity and sports; these studies have revealed positive and significant correlations between self-efficacy and autonomous motivation and controlled motivation (Nicholls et al., 2015; Sari, 2015; Neace et al., 2020), and negative correlations between self-efficacy and amotivation (Nicholls et al., 2015).

According to Roberts (2009), when individuals are gritty, their motivation increases, and this helps them to reduce obstacles or even overcome them. Previous studies have examined the relationship between grit personality and motivation and found a positive relationship between intrinsic motivation and gritty individuals (Muenks et al., 2018; e.g., Lozano-Jiménez et al., 2021). For a review in the sport domain see Cormier et al. (2021).

The transtheoretical model (Prochaska and DiClemente, 1982; Prochaska and Marcus, 1994) is another theoretical framework that has been widely used to study how people shift into and maintain healthy behaviors such as physical exercise. In this model, actions are reflected so that people can modify their behavior when facing problematic behaviors that affect their life quality. This model outlines the different stages for behavioral change in people. These stages are time dimensions that indicate at which stage people who want to modify an undesirable behavior are. This model comprises five stages of change: The first stage is pre-contemplation: an absence of behavior and the non-existent intention of making a change toward any potential issue. The second stage is contemplation: the person intends to make a change within the next 6 months. Then, during the preparation stage, the person intends to change in the near future. The next stage is action; at this stage, the subject makes the most visible changes to modify their behavior. Finally, the maintenance stage is a state in which the person is able to maintain their modified behavior (Prochaska and Marcus, 1994).

In order to determine the degree of readiness to start a behavioral shift, some authors have proposed the readiness to change index. The readiness to change index has been used as an important variable in predicting the long-term success of weight management (Dixon et al., 2009). Readiness to change refers to the degree to which an individual is motivated to change problematic behaviors and implies willingness or behavioral readiness to initiate a behavioral shift (Dunn et al., 2003). For example, people going to gyms may seem ready to start an active lifestyle, but they might not have actually embraced the effort and discipline that this activity requires. Some of these people may have been compelled by family or friends to exercise, but they are not ready to change their habits yet. The readiness to change index is calculated by subtracting pre-contemplation from the sum of the contemplation, action, and maintenance scores (Ghannadiasl et al., 2014). Several studies have examined the relationships among the types of motivation and how these relate to the stages of change toward exercise. These studies have found positive correlations with motivations (autonomous and controlled), and negative correlations with amotivation. It is also noteworthy that less self-determined regulations (external and introjected) and amotivation prevail during the pre-contemplation and contemplation stages, but decrease during the action and maintenance stages. On the other hand, the more self-determined regulations (identified, integrated and intrinsic) prevail during the action and maintenance stages, and have less dominance during the pre-contemplation and contemplation stages (Matsumoto and Takenaka, 2004; Zamarripa et al., 2018).

Other studies (e.g., Reed et al., 2013) have examined the relationship between grit and the stages of change of the transtheoretical model (TM) indicating that higher scores on Grit were associated with higher TM stage levels for moderate and high-intensity exercise, whereas grit was not associated with low-intensity TM stage. Previous studies examining the relationship between self-efficacy and stages of change showed that self-efficacy ratings increased with each progressive stage of change, supporting previous studies that showed that adherence to exercise was better maintained in participants with high self-efficacy (Simonavice and Wiggins, 2008).

In Hermosillo, Sonora, 49.7% of the population aged 20 or older suffers from obesity, whereas 35.8% of the population does not engage in physical activity due to a lack of motivation (Ensanut, 2016). It is necessary that physical education teachers, coaches, directors and health-promoting government agencies achieve a deep understanding that motivation is a fundamental tool that should be used in physical activity programs so that more people adhere to this practice (Standage et al., 2005). The results will provide information on how to develop intervention programs for adherence to physical exercise focused on developing and strengthening grit personality and self-efficacy. These programs will help to develop a higher quality motivation so that people have a greater predisposition to shift from sedentary behaviors into a more active and healthier lifestyle. Similarly, benefits in terms of physical, mental, social and economic health can also be obtained.

**Figure 1:**
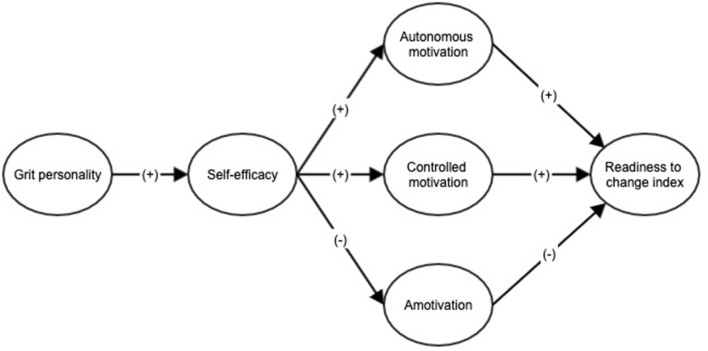
Hypothesized structural equation model of the relationship between grit personality, self-efficacy, motivation (autonomous, controlled, and amotivation) and readiness to change index.

Based on the previous studies and the relationship between the different variables, the aim of this study was to examine the relationship between grit personality, self-efficacy, autonomous motivation, controlled motivation, amotivation and the readiness to change index toward exercise in the adult population. In addition, the following hypotheses were established (see Figure 1). H1: Grit personality is expected to be positively correlated with self-efficacy, and this in turn to be positively correlated with autonomous motivation and with the readiness to change index toward exercise. H2: Grit personality is expected to be positively correlated with self-efficacy, and this in turn to be positively correlated with controlled motivation, and with the readiness to change index toward exercise. H3: Grit personality is expected to be positively correlated with self-efficacy, and this in turn to be negatively correlated with amotivation and, in the same way, with the readiness to change index toward exercise.

## Materials and Methods

### Type of Study

This study is an empirical research of associative strategy with an explanatory, cross-sectional purpose with latent variables (Ato et al., 2013).

### Population and Sample Size

Sampling was non-probabilistic for convenience. The total sample consisted of 391 adults aged between 18 and 64 years old (*M* = 31.16; *SD* = 12.45) from Hermosillo, Sonora Mexico, of which 47.8% were men and 52.2% women, complying with the minimum sample size recommended (Catena et al., 2003; Stevens, 2009; Hair et al., 2014). The inclusion criterion considered the ages ranging from 18 to 64 years, as this range is the one used to refer to the adult population (World Health Organization, 2018). Similarly, residents of Hermosillo, Sonora were chosen because of the high percentage of obesity among the adult population of this city (Ensanut, 2016).

### Procedures

The different questionnaires of the variables to be studied were combined in an instrument created using Google Forms. The participants were advised of the purpose of the study and that their data would remain confidential. Once the instrument was prepared, a pilot study was conducted (*n* = 30; 14 men, 16 women) on subjects aged between 19 and 49 years old (*M* = 25.57, *SD* = 7.17) to monitor possible errors and doubts. Adjustments were made taking into account the feedback from the examinees and the experts. Finally, the revised Google Forms instrument was applied in different areas of Hermosillo, Sonora.

The study was conducted according to the guidelines of the Declaration of Helsinki, and ethical review and approval were waived for this study because at the time the research was conducted it was not necessary to request approval from the Ethics Committee of the University of Sonora (Mexico). Participants provided their written informed consent to participate in this study and none refused to participate.

### Measurements

Grit personality was assessed using the Mexican Spanish version by Marentes-Castillo et al. (2019) of the Grit Personality Scale by Duckworth et al. (2007). This scale consists of 12 items split into six items measuring consistency of interest (e.g., “I often set a goal but later choose to pursue a different one”), and six items measuring persistence of effort (e.g., “I have achieved a goal that took years of work”). The questionnaire begins with the heading “On a scale from 1 to 5, how much do you relate to the following descriptions….” Answers are given on a Likert scale ranging from 1 (not at all) to 5 (very much). The scale has shown acceptable reliability and validity (Duckworth et al., 2007; Marentes-Castillo et al., 2019). In this study we used a grit composite measure instead of analyzing consistency of interest and persistence of effort separately, that is, the grit latent variable was created by calculating the mean of all the items that made up the two subscales. Confirmatory factor analysis (CFA) results revealed satisfactory goodness-of-fit indices: χ^2^ = 140.60; *df* = 53; NNFI = 0.96; CFI = 0.97; RMSEA = 0.06.

Self-efficacy was evaluated using the Mexican version (Delgado et al., 2017) of the Exercise Self-Efficacy Questionnaire (Marcus et al., 1992). This scale consists of five items preceded by a brief explanation: “Physical activity or exercise includes activities such as: brisk walking, jogging, cycling, swimming, or any other activity in which the effort is at least as intense as in these activities. Please select the number that indicates how confident you are of being physically active in each of the following situations…”; an item example is “When I'm tired.” Answers are given on a Likert scale ranging from 1 (not confident at all) to 5 (extremely confident). This questionnaire has shown adequate validity and reliability (Delgado et al., 2017). The latent variable was created by calculating the mean of all the items that made up the scale. CFA results revealed satisfactory goodness-of-fit indices: χ^2^ = 7.76; *df* = 4; NNFI = 0.99; CFI = 0.95; RMSEA = 0.04.

Motivation was measured using the Mexican version (Marentes-Castillo et al., 2019) of the Treatment Self-Regulation Questionnaire (Williams et al., 1996). This instrument consists of 15 items divided into three dimensions: autonomous motivation (6 items, e.g., “Because I feel that I want to take responsibility for my own health”), controlled motivation (6 items, e.g., “Because I would feel guilty or ashamed of myself if I did not exercise regularly”), and amotivation (3 items, e.g., “I really do not think about it”). The questionnaire is introduced by the heading: “The following sentence relates to the reasons why you would start exercising regularly or continue to do so. People do this for different reasons, and we want to know how much do you relate to each of the given reasons. The 15 reasons shown refer to the sentence inside the quotation marks. On a scale from 1 to 7, indicate how much do you relate to each reason: “The reason why I would exercise regularly is….” Responses are given on a Likert scale ranging from 1 (not at all) to 7 (very much). The questionnaire has shown acceptable validity and reliability (Marentes-Castillo et al., 2019). The latent variables were created by calculating the mean of all the items that made up the different dimensions. CFA results revealed satisfactory goodness-of-fit indices: χ^2^ = 356.41; *df* = 87; NNFI = 0.95; CFI = 0.96; RMSEA = 0.08.

The stages of change were measured using a Mexican version (Zamarripa et al., 2021) of the Stages of Change Scale for Exercise by Reed (1995), which consists of 24 items divided into six dimensions: pre-contemplation non-believer (4 items, e.g., “As far as I am concerned, I do not need to exercise regularly”), pre-contemplation believer (4 items, e.g., “I do not have the time or energy to exercise regularly right now”), contemplation (4 items, e.g., “I have been thinking that I might want to start exercising regularly”), preparation (4 items, e.g., “I have set up a day and a time to start exercising regularly within the next few weeks”), action (4 items, e.g., “I am finally exercising regularly”), and maintenance (4 items, e.g., “I have been exercising regularly for a long time and plan to continue doing so”). The instrument is presented by the heading: “Regular exercise is any planned physical activity (e.g., brisk walking, sports, aerobics, jogging, biking, swimming, rowing, etc.) with the purpose of improving fitness. This activity should be done 3 to 5 times a week for at least 20 to 60 min per session. Exercise does not need to be painful to be effective but must be done at a pace that increases your breathing rate and causes sweating. On a scale from 1 to 5, tell us how much do you agree with the following statements….” Answers are given on a Likert scale ranging from 1 (strongly disagree) to 5 (strongly agree). The instrument has shown good reliability and validity (e.g., Lerdal et al., 2008). The readiness to change index is calculated by subtracting pre-contemplation from the sum of the contemplation, action, and maintenance scores. The higher the index score the higher the readiness to change and the lower the index score the lower the readiness to change. CFA results revealed satisfactory goodness-of-fit indices: χ^2^ = 603.34; *df* = 237; NNFI = 0.98; CFI = 0.98; RMSEA = 0.06.

### Data Analysis

Data normality tests, and descriptive and scale reliability analyses were performed using the statistical program SPSS Statistics V.24.0. The internal consistency of each instrument was evaluated using Cronbach's alpha. Confirmatory Factor Analyses (CFA) were performed for each instrument; in addition, the measurement model was tested. Finally, the hypothesized structural equation model (Figure 1) was examined, using latent variables. Polychoric correlation matrices and asymptotic covariances were used as input for the analyses, using the Maximum Likelihood estimation method and the LISREL 8.80 software (Jöreskog and Sörbom, 2006). Fitness indices examined were: The Non-Normed Fit Index (NNFI), the Comparative Fit Index (CFI), and the Root Mean Square Error of Approximation (RMSEA). NNFI and CFI values above 0.90 indicate an acceptable data fit (Hu and Bentler, 1995), whereas RMSEA values below 0.08 are considered optimal (Cole and Maxwell, 1985).

## Results

### Descriptive Statistics, Reliability, and Bivariate Correlations

Table 1 shows descriptive statistics (range, means, standard deviations, skewness, kurtosis, and Cronbach's alphas), Participants' responses showed that values for the grit personality, self-efficacy, and autonomous motivation were above the mean value of the questionnaire, while controlled motivation and amotivation were under the mean value. In relation to the stages of change, participants' reported values in pre-contemplation, and preparation stages were below the mean value of the questionnaire, whereas contemplation, action and maintenance stages were above this value. The descriptive results of the scales revealed values between 2 and −2 for skewness and kurtosis, indicating a normal distribution of data (Muthén and Kaplan, 1985, 1992; Bandalos and Finney, 2010). The internal consistency analyses for the instruments revealed acceptable Cronbach's alpha values above 0.70, with the exception of the amotivation subscale and the pre-contemplation non-believers stage subscale. Nevertheless, as they are composed of a small number of items and have no diagnostic purpose, these scales are still considered acceptable (Nunnally and Bernstein, 1967; Schmitt, 1996; Graham, 2006; Dall'Oglio et al., 2010) (see Table 1).

**Table 1:** Descriptive statistics, and internal consistency, of the study variables.

**Variable**	**Range**	**Mean**	**SD**	**Skewness**	**Kurtosis**	**Alpha**
Grit personality	1–5	3.34	0.71	−0.71	−0.10	0.82
Self-efficacy	1–5	3.11	0.95	−0.26	−0.62	0.75
Autonomous motivation	1–7	6.05	1.14	−1.78	3.58	0.89
Controlled motivation	1–7	2.96	1.24	0.47	−0.30	0.73
Amotivation	1–7	2.52	1.40	0.89	0.24	0.51
Precontemplation non-believer	1–5	1.96	0.88	0.84	0.40	0.53
Precontemplation believer	1–5	2.62	1.27	0.28	−1.04	0.87
Contemplation	1–5	4.02	0.93	−1.03	0.74	0.76
Preparation	1–5	2.82	1.21	0.21	−1.00	0.80
Action	1–5	3.34	1.33	−0.43	−1.07	0.92
Maintenance	1–5	3.28	1.31	−0.28	−1.09	0.89
Readiness to change index	−5.25–13	6.06	4.10	−0.31	−0.63	–

Finally, most of the variables were significantly correlated and in line with expectations (see Table 2). The self-efficacy and controlled motivation variables were not related to each other, as expected. The relationships between grit, self-efficacy and autonomous motivation, and the readiness to change index toward exercise were high and positive.

**Table 2:** Correlations between study variables.

**Variable**	**1**	**2**	**3**	**4**	**5**	**6**	**7**	**8**	**9**	**10**	**11**
1. Grit personality	–										
2. Self-efficacy	0.49^**^	–									
3. Autonomous motivation	0.38^**^	0.31^**^	–								
4. Controlled motivation	−0.28^**^	−0.02	−0.14^**^	–							
5. Amotivation	−0.30^**^	−0.26^**^	−0.50^**^	0.42^**^	–						
6. Pre-contemplation non-believer	−0.29^**^	−0.18^**^	−0.40^**^	0.15^**^	0.30^**^	–					
7. Precontemplation believer	−0.32^**^	−0.34^**^	−0.31^**^	0.16^**^	0.31^**^	0.41^**^	–				
8. Contemplation	0.01	0.03	0.25^**^	0.04	−0.14^**^	−0.14^**^	0.02	–			
9. Preparation	0.12^*^	0.19^**^	0.14^**^	0.09	−0.01	−0.11^*^	−0.27^**^	0.36^**^	–		
10. Action	0.36^**^	0.47^**^	0.35^**^	−0.07	−0.28^**^	−0.27^**^	−0.61^**^	0.15^**^	0.48^**^	–	
11. Maintenance	0.41^**^	0.46^**^	0.35^**^	−0.14^**^	−0.28^**^	−0.29^**^	−0.63^**^	0.08	0.47^**^	0.84^**^	–
12. Readiness to change index	0.41^**^	0.45^**^	0.46^**^	−0.14^**^	−0.37^**^	−0.56^**^	−0.79^**^	0.32^**^	0.49^**^	0.88^**^	0.87^**^

* *p < 0.05*,

** *p < 0.01*.

### Measurement Model

Following the two-step approach suggested by Anderson and Gerbing (1988), a measurement model was examined prior to performing the structural equation model to determine whether the indicators (i.e., the latent variables items) correlate with their factors satisfactorily. The items previously discussed in the CFA sections of the instruments serve as indicators for all latent variables as appropriate. The goodness-of-fit indices of the measurement model were satisfactory (χ^2^ = 1,431.62, *p* < 0.001, *df* = 454, NNFI = 0.93, CFI = 0.94, RMSEA = 0.07).

### Structural Equations Modeling

**Figure 2:**
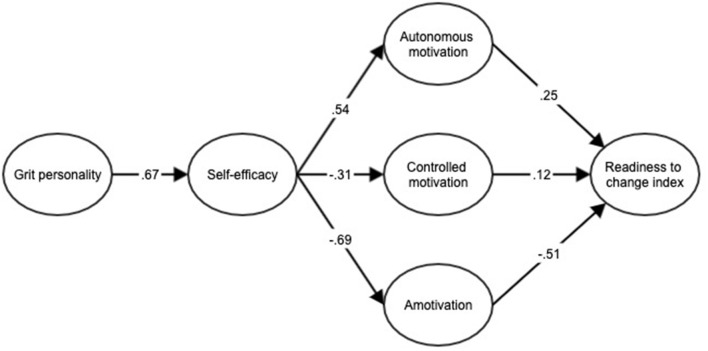
Standardized solution of the hypothesized structural model of the relationship between grit personality, self-efficacy, motivation, and readiness to change index. All regression coefficients were significant (*p* < 0.05). This figure does not include observed variables for clarity of presentation.

The hypothesized model was tested (Figure 1) as the second step of the Anderson and Gerbing approach (1988). The goodness-of-fit indices of the structural equations model were satisfactory (χ^2^ = 1,742.86, *p* < 0.001, *df* = 489, NNFI = 0.92, CFI = 0.92, RMSEA = 0.08). The structural equations model (see Figure 2) showed that grit personality was positively correlated with self-efficacy, whereas self-efficacy had positive correlations with autonomous motivation and with the readiness to change index. On the other hand, self-efficacy was negatively correlated with controlled motivation, which was positively correlated with the readiness to change index. Finally, self-efficacy was negatively correlated with amotivation, and this in turn with the readiness to change index.

In addition, the model revealed indirect effects of grit personality on autonomous motivation (β = 0.36), controlled motivation (β = −0.21) and amotivation (β = −0.47) through self-efficacy.

## Discussion

This study aimed to test a structural equation model in which the relationships among grit personality, self-efficacy, different types of motivation, and the readiness to change index toward exercise were examined in a sample of adult population. Overall, the results provided support for the hypothesized model confirming most of the proposed relationships. Results are discussed according to the established hypotheses.

Hypothesis number one was confirmed as grit personality was positively correlated with self-efficacy, which in turn was positively correlated with autonomous motivation and with the readiness to change index toward exercise. These results are in line with findings by other authors such as Ciaccio (2019), who found positive relationships between grit personality and self-efficacy. Similarly, other studies confirm positive relationships between self-efficacy and autonomous motivation, asserting the idea that the more efficacy individuals see in themselves, the higher level of motivation they will have to achieve their goals (Sari, 2015; Neace et al., 2020). Regarding the types of motivation (autonomous, controlled, and amotivation) and the readiness to change index, the results of the study by Matsumoto and Takenaka (2004) presented similarities with those presented here, since positive relationships were found between autonomous motivation and the readiness for change index. The results of Zamarripa et al. (2018) shared similarities as well by showing that people who were at the maintenance stage presented higher levels of autonomous motivation.

Further results revealed a negative relationship between self-efficacy and controlled motivation, and because a positive correlation between self-efficacy and controlled motivation was expected, hypothesis number two is rejected. However, some studies (Neace et al., 2020) have found a positive relationship between self-efficacy and controlled motivation with a sample of university students rather than the older age range sample examined in this study. The results obtained may stem from the fact that the higher the quality of a person's motivation (autonomous motivation), the greater their affinity for the active stages, that is, for the action stage and the maintenance stage. On the other hand, the lower the quality of motivation (controlled motivation and amotivation), the more a person will relate to inactive stages such as pre-contemplation and contemplation (Zamarripa et al., 2018).

Hypothesis number three is deemed confirmed according to the results of the structural equation model. Grit personality was positively correlated with self-efficacy, which in turn was negatively correlated with amotivation and, likewise, was negatively correlated with the readiness to change index toward exercise. The results of this research are in line with the results obtained by Nicholls et al. (2015) using a sample of athletes as opposed to the sample of this study composed of adults from the general population. In addition, the study by Zamarripa et al. (2018) found that amotivation is more predominant at pre-contemplation stages. Our findings are consistent with the assumptions proposed by self-determination theory and with previous research (e.g., Daley and Duda, 2006) where it was shown that higher self-determination appeared to be a prerequisite for regular exercise practice and classification in one of the higher stages of readiness to change for exercise, and thus lower self-determination (amotivation) would be placed in the lower levels of readiness to change. People who do not have a clear motive for being physically active perceive themselves as less prepared to make attempts to change their sedentary behavior. Amotivation occurs when individuals are not clear about their motives for performing a behavior, and are unable to foresee the consequences of their behaviors, have doubts about their actions and are likely to desist in the future, this being the main characteristic of precontemplators, people who do not act and have no intention of acting shortly (Prochaska et al., 1992).

### Limitations and New Lines of Research

This study faces some limitations as well, for example, its design and type of study; since cross-sectional studies do not allow to establish causal relationships, it would be convenient to conduct a longitudinal or experimental study with the same variables and a determined population. Another limitation is the non-probabilistic convenience sampling method, so it is suggested to carry out studies with stratified random sampling, as well as with a larger sample, to ascertain the behavior of the people of Hermosillo in regard to the examined variables with greater accuracy.

## Conclusion

Grit is a personality trait that has a positive effect on self-efficacy; namely, it contributes positively to the belief that people have about their own ability to perform physical exercise on a regular basis. Perhaps due to the component of passion and perseverance that characterizes grit and the perception of competence provided by self-efficacy, this promotes higher-quality motivational regulations (intrinsic, integrated, and identified) and contributes to making people more willing to embrace or to maintain a regular exercise behavior. In addition, grit and self-efficacy limit the development of lower-quality motivational regulations (introjected, external, and amotivation), which were correlated with a lower disposition to change sedentary behaviors.

Despite the numerous benefits of regular physical exercise, only 42.1% of Mexican adults are physically active, with only half of them (54.8%) meeting the current recommendations for healthy physical activity (INEGI, 2020). Therefore, it is imperative to design procedures based on psychological theories, such as the ones used in this study, to effectively promote the adoption and maintenance of active behaviors and lifestyles by the nearly 60 million sedentary Mexicans. Because developing a grit personality bolsters confidence in one's ability to exercise regularly, as well as higher-quality motivation and a willingness to shift from sedentary behaviors into a more active and healthier lifestyle, the results of this study provide valuable information with regard to these programs.

## Data Availability Statement

The original contributions presented in the study are included in the article, further inquiries can be directed to the corresponding author.

## Ethics Statement

Ethical review and approval was not required for the study on human participants in accordance with the local legislation and institutional requirements. The participants provided their written informed consent to participate in this study.

## Author Contributions

MD, JZ, and IC: conceptualization, formal analysis, investigation, methodology, and supervision. AZ: data curation. MD: funding acquisition, project administration, and resources. MD, AZ, JZ, IC, AB, HD, and KV: writing—original draft, writing—review, and editing. All authors have read and agreed to the published version of the manuscript.

## Conflict of Interest

The authors declare that the research was conducted in the absence of any commercial or financial relationships that could be construed as a potential conflict of interest.

## Publisher's Note

All claims expressed in this article are solely those of the authors and do not necessarily represent those of their affiliated organizations, or those of the publisher, the editors and the reviewers. Any product that may be evaluated in this article, or claim that may be made by its manufacturer, is not guaranteed or endorsed by the publisher.
